# Supplementary material for: Patterns of Intron Gain and Loss in Fungi
Source: PLoS Biol. 2004 Nov 30;2(12):e422. doi: 10.1371/journal.pbio.0020422 (PMC532390; doi:10.1371/journal.pbio.0020422)
Supplement: Table S1 — Also available at http://genes.mit.edu/NielsenEtAl/. (4.3 MB ZIP). [file pbio.0020422.st001.zip › NielsenEtAl/html/260.html]

AN7721.1.NCU08897.1.MG04856.1.FG09016.1


```
 CLUSTAL W (1.82) Multiple Sequence Alignments - Introns Inserted


Sequence 1: MG04856.1	477 aa
Sequence 2: FG09016.1	468 aa
Sequence 3: NCU08897.1	476 aa
Sequence 4: AN7721.1	478 aa
Alignment Length: 479 aa
Number Identitical Residues: 381 aa
Alignment Score (without introns) 15557


MG04856.1 	MCATV~RFLDLVKPFVPFLPEVQQPETKIPFNQKLMWTG~LTLLIFLVMSQMPLYGIVSS
NCU08897.1	-MSSL1RFLDLVKPFVPFLPEVQQPETKIPFNQKLMWTG~LTLLIFLVMSQMPLYGIVSS
FG09016.1 	-----~----MVKPFVPFLPEVQQPETKIPFNQKLMWTA~LTLLIFLVMSQMPLYGIVSS
AN7721.1  	-MSGL1RFLDLIKPFTPLLPEVAAPETKVPFNQKLMWTG0LTLLIFLVMSQMPLYGIVSS
          	  :      .::***.*:****  ****:*********. ********************

MG04856.1 	DTSDPLYWLRMMMASNRGTLMELGITPIISSGMVFQ~LLAGTHMIDVNLDLKSDRELYQT
NCU08897.1	DTSDPLYWLRMMMASNRGTLMELGITPIISSGMVFQ~LLAGTHMIDVNLDLKADRELYQT
FG09016.1 	DNSDPLYWLRMVIASNRGTLMELGITPIISSGMVFQ~LLAGTHMIDVNLDLKSDRELYQT
AN7721.1  	DTSDPLYWLRMMLASNRGTLMELGITPIISSGMVFQ0LLAGTHLIDVNLDLKTDRELYQT
          	*.*********::*********************** ******:********:*******

MG04856.1 	AQKLFAFILSAGTATVYVFTGLYGPPSELGAGIVFLLILQLFIAGMIVILLDELLQKGYG
NCU08897.1	AQKLFAVILSIGTATVYVFTGLYGPPSDLGAGIVFLLILQLVVAGMIVILLDELLQKGYG
FG09016.1 	AQKLLAFILSAGTATVYVFSGLYGPPSDLGAGIVFLLILQLVVAGMIVILLDELLQKGYG
AN7721.1  	AQKLFAIILSFGQACVYVLTGLYGQPSDLGAGICVLLIVQLVVAGLVVILLDELLQKGYG
          	****:*.*** * * ***::**** **:***** .***:**.:**::*************

MG04856.1 	LGSGISLFIATNICESIMWKAFSPTSINTGRGPEYEGAVIALFHLLMTWPNKQRALQEAF
NCU08897.1	LGSGISLFIATNICESIMWKAFSPTSINTGRGPEYEGAVIALFHLLMTWDNKQRALYEAF
FG09016.1 	LGSGISLFIATNICESIMWKAFSPTTINTGRGPEFEGAVIALFHLLFTWPNKQRALQEAF
AN7721.1  	LGSGISLFIATNICESIVWKAFSPTTINTGRGPEFEGAIIALFHLLFTWSDKQRALREAF
          	*****************:*******:********:***:*******:** :***** ***

MG04856.1 	YRQNLPNIMNLLATLLVFCAVIYLQGLRVEIPVKSSRQRGARGSYPVRLFYTSNMPIMLQ
NCU08897.1	YRQNLPNIMNLLATLVVFAAVIYLQGFRVEIPVKSSRQRGARGSYPIRLFYTSNMPIMLQ
FG09016.1 	YRQNLPNIMNLLATIAVFAAVIYLQGFRVEIPVKSSRQRGARGSYPVRLFYTSNMPIMLQ
AN7721.1  	YRQNLPNVMNLLATLVVFAAVIYLQGFRVEIPVKSSRQRGMRGSYPVRLFYTSNMPIMLQ
          	*******:******: **.*******:************* *****:*************

MG04856.1 	SALSSNIFIISQMLYSRFPENLLVRLFGVWEAKEGSSQLSAISGLTYYMSPPMNFTEALI
NCU08897.1	SALSSNVFLISQMLYSRFSENLLVRLFGVWEAKEGTAQLSAVSGLVYYMSPPLNFKDALL
FG09016.1 	SALSSNVFLISQMLYSRFSENLLVRLFGVWEAKDGSSQLHAASGLVYYMSPPQSMKDALL
AN7721.1  	SALCSNIFLISQMLYSRFSDNILVKLLGVWEPREGSAQLHASSGVAYYMSPPLNFREALL
          	***.**:*:*********.:*:**:*:****.::*::** * **:.****** .: :**:

MG04856.1 	DPIHTALYIVYMLTACAVFSKTWIEVSGSSPRDVAKQLKDQGLVMAGHRDQSMYKELKRI
NCU08897.1	DPIHTAVYIAYMLTACAVFSKTWIEVSGSSPRDVAKQLKDQGLVMAGHREQSMYKELKRI
FG09016.1 	DPIHMSAYIIYMLGACALFSKTWIEVSGSSPRDVAKQLKDQGLVMAGHRDQSMYKELKRI
AN7721.1  	DPIHTAVYITFMLVACALFSKTWIEVSGSAPRDVAKQLKDQGLVMAGHREQSMYRELKRI
          	**** : ** :** ***:***********:*******************:****:*****

MG04856.1 	IPTAAAFGGACIGALSVASDLMGALGSGTGTLLAVT2IIYGYFEIAAKEGDLAG-MKGMV
NCU08897.1	IPTAAAFGGACIGALSVASDLMGALGSGTGTLLAVT2IIYGYFEIAAKEGDLQG-MKGMI
FG09016.1 	IPTAAAFGGACIGALSVTSDLLGALGSGTGTLLAVT2IIYGYFEIAAKEGDMSG-MKGMI
AN7721.1  	IPTAAAFGGACIGALSVASDLLGALGSGTGILLAVT2IIYGYFEIAAREGDIGGGLKGLV
          	*****************:***:******** ***** **********:***: *.:**::

MG04856.1 	MG-
NCU08897.1	MG-
FG09016.1 	MG-
AN7721.1  	PGN
          	 *.
```
